# Emergency Department Computed Tomography Use for Non-traumatic Abdominal Pain: Minimal Variability

**DOI:** 10.5811/westjem.2018.6.37381

**Published:** 2018-07-26

**Authors:** Roderick Cross, Rahul Bhat, Ying Li, Michael Plankey, Kevin Maloy

**Affiliations:** * Georgetown University Hospital/Washington Hospital Center, Department of Emergency Medicine, Washington, District of Columbia; † Georgetown University Medical Center, Department of Medicine, Washington, District of Columbia

## Abstract

**Introduction:**

Variability in the use of computed tomography (CT) between providers in the emergency department (ED) suggests that CT is ordered on a provider rather than a patient level. We aimed to evaluate the variability of CT ordering practices for non-traumatic abdominal pain (NTAP) across physicians in the ED using patient-visit and physician-level factors.

**Methods:**

We conducted a retrospective study among 6,409 ED visits for NTAP from January 1 to December 31, 2012, at a large, urban, academic, tertiary-care hospital. We used a two-level hierarchical logistic regression model to estimate inter-physician variation. Intraclass correlation coefficient (ICC) was calculated.

**Results:**

The hierarchical logistic regression analyses showed that patient-visit factors including younger age, arrival mode by ambulance, prior CT, >79 ED arrivals in the previous four hours, and ultrasound had statistically significant negative associations with physician CT ordering, while surgical team admission and white blood count (WBC) >12.5 K/millimeter cubed (mm^3^) had statistically significant positive associations with physician CT ordering. With physician-level factors, only physicians with >21 years experience after medical school graduation showed statistical significance negatively associated with physician CT ordering. Our data demonstrated increased CT ordering from the mean in only one out of 43 providers (2.3%), which indicated limited variation across physicians to order CT. After adjusting for patient-visit and physician-level factors, the calculated ICC was 1.46%.

**Conclusion:**

We found minimal physician variability in CT ordering practices for NTAP. Patient-visit factors such as age, arrival mode, admission team, prior CT, ED arrivals in previous four hours, ultrasound, and WBC count were found to largely influence CT ordering practices.

## INTRODUCTION

Computed tomography (CT) utilization in the emergency department (ED) has increased significantly in the past 30 years.^1^ A 330% rise was observed from 1996 to 2007 in a retrospective study of the National Hospital Ambulatory Medical Care Survey, with utilization for non-traumatic abdominal pain (NTAP) representing the highest growth rate in CT use.^2^ During this period, abdominal pain composed 6.5% of total ED visit chief complaints, with related CT usage increasing from 1.4% in 1996 to 33% in 2005–2007.^2^–^4^ However, rates can be as high as 45%–50% when considered in high-risk groups.^1^

Studies measuring CT use and associated outcomes and ordering practices for NTAP have not been in agreement. Rates of change in diagnosis and change in disposition for NTAP in five studies have been as high as 54% and 40%, respectively.^5^–^9^ Yet three studies describe an increase in diagnostic specificity for NTAP but with no change in admission rates, missed surgical diagnoses, or six-month mortality.^10^–^12^ One study showed minimal variability in physician ordering practices when examining overall CT use, while another showed significant variability when examining exclusively NTAP CT use.^13^–^15^

Increased CT use adds additional costs to clinical evaluation and treatment. Furthermore, concerns related to radiation exposure and the risks of benign, incidental findings are legitimate.^16^,^17^ Within this context of equivocal risk-benefit and cost-benefit understanding, examination of variability in CT ordering practices across physicians, as well as against physician level and patient-visit level predictive factors will contribute to the identification of appropriate use^18^–^20^ and may suggest guideline modifications that could result in decreased imaging with similar or improved outcomes. We examined the variability of CT ordering practices for NTAP across physicians in the ED using both patient-visit and physician-level factors. This focus adds to previously published literature, which has predominantly examined physician-level factors only or overall CT use, respectively.

## METHODS

### Study Design and Data Collection

We conducted a retrospective cohort design, reviewing the electronic medical records (EMR) of patients visiting the ED at a large, urban, academic, tertiary-care hospital. EMR patient visit-level data included demographics, dates and times of ED registration, discharge and admission, diagnosis, attending physician, dates and times of image order, test name and results. The physician’s gender and education background was extracted from the public-access hospital website. This study was approved by the institutional review board with informed consent waiver and was compliant with the Health Insurance Portability and Accountability Act (HIPAA).

**Figure 1:**
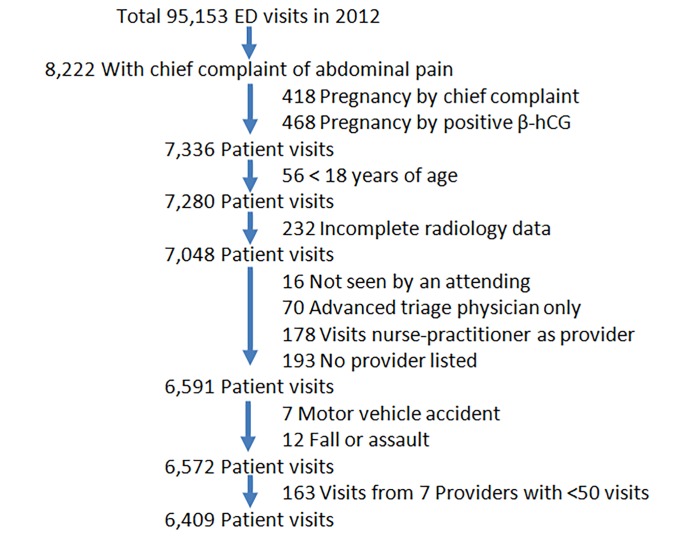
Flow chart of sampling procedure for excluding patient visits from a study on use of computed tomography for chief complaint of non-traumatic abdominal pain.

We included all patient visits from January 1 to December 31, 2012, with a chief complaint of abdominal pain. We excluded patient visits from the trauma unit as well as those with pregnancy, patients less than 18 years old, with attending physician’s annual NTAP visits < 50 (similar to Levine et al.),^15^ with incomplete radiology data, without attending physician, or any visit associated with trauma. See Figure 1 for the detailed exclusions.

**Population Health Research Capsule**

What do we already know about this issue?

Studies examining computed tomography (CT) use among emergency physicians for overall use and non-traumatic abdominal pain (NTAP) have demonstrated minimal and significant variability, respectively.

What was the research question?

This study evaluated the variability of CT ordering practices for NTAP among emergency physicians.

What was the major finding of the study?

The use of CT by emergency physicians for NTAP showed minimal variability and was influenced by patient-visit factors.

How does this improve population health?

Findings contribute to evidence to further clarify CT appropriate use to optimize resource utilization.

### Outcome Measure and Predictor Variables

The primary outcome was whether a physician ordered a CT during a patient’s ED visit for NTAP. We investigated both patient-visit and physician-level factors as predictor variables. Patient-visit factors included patient gender; age; arrival mode (walk-in, ambulance, or indeterminate); acuity (determined using Emergency Severity Index [ESI] – most severe, more severe, severe, less severe, or least severe); arrival time (weekday vs. weekend, and by shift – day, evening, or night); disposition (discharge, admit, observation, against medical advice/absent without leave/left without being seen, or indeterminate); admission team (surgical team, non-surgical team, or not admitted);whether or not the patient had a prior CT abdomen/pelvis; current ED volume (evaluated by counting the number of ED arrivals in the previous four hours); whether or not there was use of diagnostic ultrasound; first white blood count (WBC) count; first hemoglobin count; and first hematocrit count. Physician-level predictor variables included gender, years since completing medical school, whether or not a physician completed a fellowship, whether or not there was involvement of advanced triage (a provider with ability to initiate orders prior to full evaluation), and annual ED visit volume (sum of patient visits supervised by each physician throughout 2012). The numeric variables (i.e., ED arrivals in previous four hours, WBC count, hemoglobin count, hematocrit count, and physician’s annual ED visit volume) were all categorized into quartiles.^13^

### Data Analysis

We conducted preliminary analyses to summarize patient-visit and physician-level characteristics by CT ordering status. Univariate and multivariate generalized linear models with repeated measures were performed to investigate the associations of patient-visit and physician-level factors, respectively. We applied the iterative fitting algorithm for repeated measures in modeling to avoid the violation of the assumption of independence due to the multiple patient visits cared for by the same physician. We used a two-level hierarchical logistic regression model with physician-specific random intercepts developed by Dr. Sistrom^13^ to study the association of CT ordering with patient-visit and physician-level factors. The estimated physician-specific intercepts and associated standard errors were transformed by exponentiation to get the adjusted odds ratios with 95% confidence intervals (CI) for each physician.

To estimate the proportion of total variation attributable to the physician level after adjusting for the patient-visit and physician covariates, we calculated the intraclass correlation coefficient (ICC) by using the estimated variance of the physician-specific intercepts from the two-level hierarchical logistic regression model and an estimate of the standard logistic function variance of π^2^/3. We also calculated a reliability estimate for each physician using the formula, OIV/(OIV+SEPI)^2^, where OIV is the overall intercept variance, SEPI is the standard error for each physician, and both are produced directly from the multilevel model. The aggregate reliability with 95% CI was produced by averaging the reliability estimate for each physician. We performed all analyses using SAS v.9.4 (SAS Institute, Carry, NC, USA), and statistical significance was evaluated at the 0.05 level.

## RESULTS

Of 95,153 total ED patient visits from January 1 to December 31, 2012, 8,222 visits were for NTAP by chief complaint. After the exclusions of 418 visits with pregnancy by chief complaint and 468 by positive beta-human chorionic gonadotropin (β-hCG), 56 visits from patients less than 18 years old, 232 visits with incomplete radiology data, 457 visits without an attending physician, 19 visits associated with trauma., and another 163 visits supervised by seven providers with less than 50 annual visits, the final study population comprised 6,409 patient visits. Figure 1 shows the flow chart of sampling in detail.

Table 1 shows the demographic and clinical characteristics of the sampled patient visits. The majority were female (67.2%), 23–63 years old (73.6%), walk-ins (77.6%), during weekdays (74.9%), with moderate acuity (70.5%), were discharged from the ED (72.2%), and with no/intermediate advanced triage (76.2%). Overall, the percentage of CT ordering was 27.6% (1,770 of 6,409). After the stratification of CT ordering status, the patient visits with an ordered CT compared to those without a CT showed higher percentages in the older age group ≥ 44 years (62.9% vs. 45.0%), severe or higher acuity (95.0% vs. 81.5%), admit or observation disposition (42.1% vs. 19.6%), and admission by surgical team (15.6% vs. 4.5%).

In addition, over one third of patient visits without CT did not have the lab/record of a WBC count, hematocrit, and hemoglobin, while over 96% among the patient visits with CT ordering had these records. During the study period, 43 physicians saw the sampled ED visits. Table 2 shows the characteristics of these physicians. Over 50% of them had 10 years or longer experience after completing medical school. Over 70% of the physicians did not complete a fellowship. These physicians provided care with the median annual NTAP visit volume of 138 (interquartile range [IQR]: 97–209), and median CT ordering rate of 27.1% (IQR: 22.9–30.5%).

Table 3 shows the unadjusted and adjusted odds ratios of CT ordering for the patient-visit variables. The univariate analyses showed that CT ordering was statistically significantly higher in the patients who were male, older, with severe or higher acuity, admitted by surgical team, had a WBC count >12.5 K/mm^3^, hematocrit count >45%, and hemoglobin count >17.1 g/dL. In the multivariate model, compared to the patients aged 44–63 years old, the odds of CT imaging for younger patients significantly decreased 16–36%, but increased over 35% for older patients; the patients who arrived by ambulance (vs. walk-in) (odds ratio [OR] [0.75]; 95% CI [0.65–0.87]; P < 0.001), having prior CT imaging (OR [0.44]; 95% CI [0.30–0.65]; P < 0.001), receiving an ultrasound evaluation during visit (OR [0.71]; 95% CI [0.58–0.87]; P < 0.001), and arrived during the busiest ED periods (OR [0.82]; 95% CI [0.68–0.99]; P = 0.04) were less likely to have a CT.

The patients admitted by a surgical team were more likely to have a CT (OR [1.84]; 95% CI [1.43–2.37]; P < 0.001). WBC count was positively associated with CT ordering, where a first WBC count of > 15.5 K/mm^3^ demonstrated increased odds of CT ordering (OR, [2.24]; 95% CI [1.66–3.03]; P < 0.001). Table 4 shows that physicians who had >21 years of experience (vs. 10–21 years) after medical school (OR [0.60]; 95% CI [0.39–0.93]; P = 0.02), or completed fellowship training (OR [0.70]; 95% CI [0.53–0.92]; P = 0.01) were significantly less likely to order a CT.

In the final multilevel model, we included all patient-visit and physician-level factors together with physician-specific random effect. Table 5 shows the results of each of the patient-visit and physician-level variables; Table 6 shows only those variables that were statistically significant. The patient-visit variables showed similar associations as those in the multivariate analysis above, whereas among physician-level variables, only physicians who had >21 years of experience after graduation from medical school showed statistical significance and these physicians were less likely to order CT (OR [0.68]; 95% CI [0.48–0.96]; P = 0.03) compared to those with 10–21 years experience.

**Figure 2:**
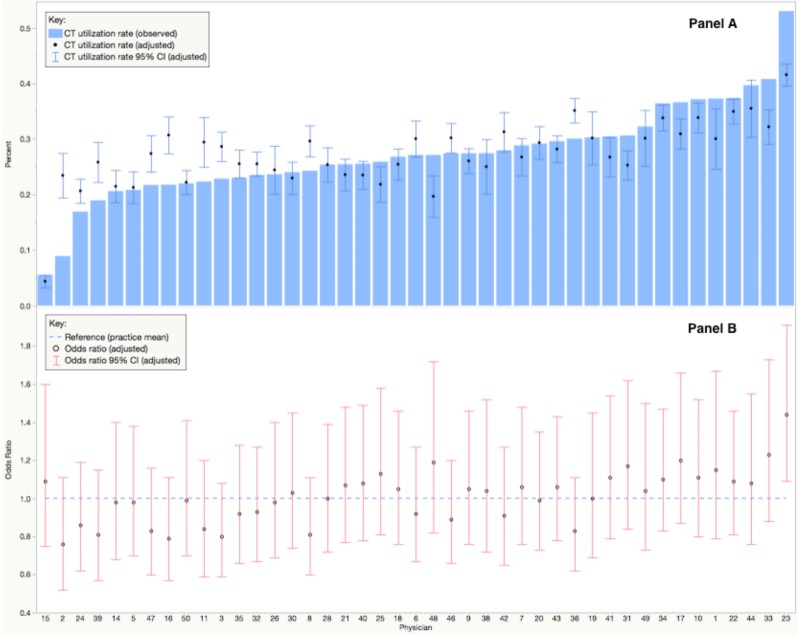
A) Observed and predicted computed tomography (CT) ordering percentage for each physician; B) Estimated odds ratio of each physician for the tendency to order CT. (All predicted and estimated values were from the hierarchical model.) *CI,* confidence interval.

Figure 2A shows the observed and predicted CT ordering rates for individual physicians plotted in ascending observed order. The predicted CT ordering rates accounted for fixed patient-visit and physician-level variables, but not for the random physician-specific intercepts. Figure 2B shows the corresponding physician-specific odds with 95% CIs for CT ordering. ORs less than one indicated the physician was less likely to order a CT; and ORs greater than one indicated higher tendency. There was only one out of 43 physicians (2.3%) with the 95% CI of OR not intersecting one, which indicated limited variation across physicians to order CT.

In the reduced model including physician-specific random intercept only, the calculated ICC was 4.73%. After adding the patient-visit and physician-level variables, the ICC was reduced to 1.46%. The estimate of reliability of the physician-specific intercepts was 0.62 (95% CI [0.61–0.64]).

## DISCUSSION

Our study found minimal physician variability in CT utilization. Moreover, numerous patient-visit factors were statistically significantly associated with CT use. While the identification of patient factors related to CT utilization is not new, our study adds to previous literature by demonstrating the overwhelming magnitude that patient-visit factors (and the minimal role that physician factors) contribute to CT ordering variability within the context of NTAP.

Both the calculated ICC and estimated reliability in our study suggested minimal physician variability in CT ordering practice, which was in accordance with the results reported by Wong et al.^13^ Specifically, “for provider profiling purposes, when reliability is above 70%, meaningful difference between some physicians (called ‘outliers’) and the mean are discernible; at 90% reliability, difference between pairs of physicians are meaningful.”^13^ Therefore, considering that our reliability was below 70%, no meaningful difference between physicians was discernible in our study. Specifically, the ICC in this study represents the percent of variability in CT ordering that could be attributed to a particular physician.

Thus, given the ICC was reduced from 4.7% to 1.46% after controlling for patient-visit factors and physician factors, two points should be highlighted. First, consideration should be given to controlling for patient-visit factors when examining resource utilization. Second, given that physicians contribute ostensibly only 1.46% to total CT use variability, care should be used when identifying outliers for overuse or underuse. Our data demonstrated increased CT ordering from the mean in one out of 43 providers. That being said, we have not overstated the provider’s difference in utilization given the minimal physician influence over CT use found in this study.

When examining physician factors separately we found years after completing medical school, fellowship, and advanced triage physician to be statistically significantly negatively associated with CT ordering. However, in the fixed-effects model considering physician and patient-visit factors jointly, only the subset of physicians with the longest period of time from completing medical school was statistically significantly negatively associated with imaging ordering, while patient-visit factors were shown to have a larger magnitude of association over CT imaging-ordering practices.

Some studies have shown that physician factors have minimal predictive value on ordering practices,^4^,^13^ which were in accordance with our results. After considering all patient-visit and physician-level factors in our multilevel analyses, most physician factors were not statistically significantly associated with CT ordering. Notwithstanding, our findings contrast with studies that have shown physician age, board certification, and risk-tolerance to have statistical significance with respect to CT ordering.^15^,^21^–^24^ Differences in population, sampling, predictors considered, and/or the sample source may explain discordance among these studies. For example, shared decision-making in academic settings may serve to dampen image-ordering provider variability, and chief complaints such as trauma or head injury may carry unique considerations related to mechanism when compared to NTAP.^21^ Conversely, elderly patient visits are associated with increased CT use due to their increased risk for abdominal pathology and their less-reliable physical exams.^25^

We found that older patients were more likely to have CT as a part of their work-up. This is consistent with the benefits of CT in diagnosing the source for NTAP in the elderly, whose clinical presentation is a diagnostic challenge.^11^ For the elderly, the etiology of NTAP often presents atypically, and abdominal tenderness or lack thereof may not be representative of the underlying pathology.^5^, ^26^

A prior CT was negatively associated with CT ordering in our study. Ostensibly, if a patient was already known to have an abdominal pathology, they may have been managed under the assumption of an acute flare of this condition, which did not require repeat imaging, in so far as their presentation is not overtly suggestive of severe progression. For example, a patient with a recently diagnosed renal or ureteral stone on CT would be unlikely to have a repeat scan as it has been shown that repeat CT in this setting does not provide additional benefit but potentially increases risk.^27^, ^28^ Moreover, if a recent CT is available, this may influence the provider to weigh concerns of radiation exposure against possible minimal added-benefit from repeat imaging in a patient with a previously negative scan or with chronic abdominal disease (e.g., a patient with inflammatory bowel disease may not receive a CT if they have recently had imaging).^29^–^31^

In our study, a radiology ultrasound performed during the patient-visit was negatively associated with CT ordering. This is consistent with previous studies, which demonstrated the ability of ultrasound to rule in or rule out pathology.^20^,^32^ While our study did not explicitly examine other imaging modalities, ultrasound potentially could make CT unnecessary in the setting of acute appendicitis or cholelithiasis.^33^,^34^ We did not evaluate emergency physician-performed bedside ultrasound. However, a bedside ultrasound that is clearly positive for cholecystitis could obviate the need for a CT.^35^ Moreover, bedside ultrasound in the setting of renal colic could similarly influence CT use.^36^

Using ED arrivals in the previous four hours as a surrogate for ED “busyness” or crowding, we found a busier ED negatively associated with CT imaging, which was different from the findings by Wong et al.^13^ This may have been due to the time required to perform a CT and obtain results. Moreover, during high-volume periods in the ED, prioritization of CT use may have taken place (consciously or unconsciously) and disposition decisions may have been based more on clinical presentation. The varying effect of ED volume and crowding has been investigated,^37^–^40^ and so impact on imaging ordering stands to reason.

Elevated WBC count was positively associated with ordering of CT. This further demonstrates the notion that patient severity would drive CT imaging. However, lack of significance of acuity represented by the ESI, while an imperfect metric,^41^ makes this picture less clear. Moreover, sensitivity and specificity of WBC counts have unclear clinical significance in isolation so clinical decision scores such as the Alvarado score and the pediatric appendicitis score take into account multiple predictors.^42^, ^43^ It should be noted that our analysis of WBC count did not examine whether the WBC count resulted before or after a CT was ordered or deferred.

It bears mentioning that the presence of an advanced triage physician did not show statistical significance. Thus, whether order sets were initiated at triage or by the physician providing direct care to the patient did not impact CT utilization. Moreover, as in other studies^13^,^15^ we did not evaluate the presence or absence of registered nurse-initiated order sets nor the possibility of resident CT ordering prior to attending consultation.

Admission to a surgical team was positively associated with CT imaging. This finding suggests that patients admitted to surgery are surgical candidates and, therefore, likely to have more severe pathology. Thus, CT imaging may be used to confirm this acuity and contribute to surgical planning.^5^, ^44^, ^45^

Arrival mode via ambulance was negatively associated with CT imaging. This was different from Wong et al.^13^ who found that arrival via ambulance was positively associated with CT imaging. Moreover, the 2010 National Hospital Ambulatory Medical Care Survey demonstrated that 73% of ambulance ED visits are for patients > 65 years old,^46^ an age group where increased use of CT was expected. While our arrival mode findings seemed contrary to that of severity driving CT imaging, one hypothesis could be that patients arriving by ambulance may have represented a disproportionate number of repeat visitors and may have had a recent CT in their medical records, which in our study was a negative predictor for CT use.

Our sample of 6,409 ED visits for NTAP was extracted from 95,153 ED visits. This is comparable to Wong et al. 13 who examined 88,851 ED visits for all types of imaging but did not provide subgroups by complaint. The subgroup of abdominal pain for Levine et al.^15^ included 18, 614 ED visits for abdominal pain, and while this robust study was three times the size of our sample, they did not account for a number of statistically significant, patient-visit factors such as prior CT, prior ultrasound, surgical admitting team, WBC count, arrival mode, and ED volume. Thus, while our study sample was smaller by comparison, our examination and identification of strongly predictive patient-visit factors adds value to current evidence.

## LIMITATIONS

Limitations to our study include error associated with data collection during patient-visits; as this was a retrospective study, we were unable to monitor the accuracy of this process. Additionally, as a single-center study within an academic setting, including resident-ordering effects, generalizability is limited beyond this context. Our study demonstrated limited variability for CT use related to NTAP exclusively. However, examination of use by all complaints may be of importance, as variability by CT modality has been observed.^15^ Furthermore, analysis of a one-year study period did not permit detection of annual trends or control of incoming or outgoing physicians. Lastly, given this was a single-center study within a single year our sample size was too small to reliably detect meaningful differences among physicians. Future research should be multicenter and multiyear to investigate the influence of patient-visit and physician-level factors on CT use.

## CONCLUSION

We found minimal physician variability in CT ordering practices for NTAP, similar to the findings by other researchers. Patient-visit factors such as age, arrival mode, admission team, prior CT, ED arrivals in previous four hours, ultrasound, and WBC count were found to largely influence CT ordering practices whereas physician-factor contributions were minimal. This study adds to previous research by uniquely quantifying the magnitude of patient-visit and physician-level factors.

## Figures and Tables

**Table 1:** Characteristics of 6,409 patient visits at emergency departments for non-traumatic abdominal pain.

Characteristic (n [%])	Without CT (n = 4639)	with CT (n = 1770)	Total (n = 6409)
Gender			
Male	1467 (31.6)	633 (35.8)	2100 (32.8)
Female	3172 (68.4)	1137 (64.2)	4309 (67.2)
Age			
18–22 yrs	581 (12.5)	89 (5.0)	670 (10.5)
23–30 yrs	936 (20.2)	220 (12.4)	1156 (18.0)
31–43 yrs	1034 (22.3)	348 (19.7)	1382 (21.6)
44–63 yrs	1490 (32.1)	689 (38.9)	2179 (34.0)
64–74 yrs	313 (6.8)	228 (12.9)	541 (8.4)
≥ 75 yrs	285 (6.1)	196 (11.1)	481 (7.5)
Arrival mode			
Walk-in	3621 (78.0)	1351 (76.3)	4972 (77.6)
Ambulance	1005 (21.7)	417 (23.6)	1422 (22.2)
Indeterminate	13 (0.3)	2 (0.1)	15 (0.2)
Acuity (Emergency Severity Index)			
Most/more severe	615 (13.3)	329 (18.6)	944 (14.7)
Severe	3164 (68.2)	1353 (76.4)	4517 (70.5)
Least/less severe	808 (17.4)	64 (3.6)	872 (13.6)
No record	52 (1.1)	24 (1.4)	76 (1.2)
Arrival time			
Monday–Friday daytime	1496 (32.2)	578 (32.7)	2074 (32.4)
Monday–Friday evening	1278 (27.6)	484 (27.3)	1762 (27.5)
Monday–Friday nighttime	688 (14.8)	274 (15.5)	962 (15.0)
Saturday–Sunday daytime	483 (10.4)	178 (10.0)	661 (10.3)
Saturday–Sunday evening	450 (9.7)	173 (9.8)	623 (9.7)
Saturday–Sunday nighttime	244 (5.3)	83 (4.7)	327 (5.1)
Disposition of patient visit			
Discharge	3617 (78.0)	1013 (57.2)	4630 (72.2)
Admit	794 (17.1)	646 (36.5)	1440 (22.5)
Observation	114 (2.5)	99 (5.6)	213 (3.3)
Against medical advice/absent without leave/left without being seen	104 (2.2)	8 (0.5)	112 (1.8)
Indeterminate	10 (0.2)	4 (0.2)	14 (0.2)
Admission team			
Non-surgical team	723 (15.6)	488 (27.6)	1211 (18.9)
Surgical team	209 (4.5)	277 (15.6)	486 (7.6)
Not admitted	3707 (79.9)	1005 (56.8)	4712 (73.5)
Advanced triage physician?			
No/indeterminate advanced triage	3588 (77.3)	1293 (73.1)	4881 (76.2)
Advanced triage	1051 (22.7)	477 (26.9)	1528 (23.8)
Prior CT abdomen/pelvis	103 (2.2)	25 (1.4)	128 (2.0)
ED arrivals in previous 4 hours			
≤42	1078 (23.2)	451 (25.5)	1529 (23.9)
>42 and ≤62	1187 (25.6)	440 (24.8)	1627 (25.4)
>62 and ≤79	1214 (26.2)	460 (26.0)	1674 (26.1)
>79	1160 (25.0)	419 (23.7)	1579 (24.6)
Ultrasound abdomen/pelvis evaluation	520 (11.2)	186 (10.5)	706 (11.0)
First white blood cell count, K/mm3			
≤3.9	143 (3.1)	68 (3.8)	211 (3.3)
>3.9 and ≤12.5	2510 (54.1)	1297 (73.3)	3807 (59.4)
>12.5 and ≤15.5	164 (3.5)	196 (11.1)	360 (5.6)
>15.5	133 (2.9)	169 (9.5)	302 (4.7)
No labs/no record	1689 (36.4)	40 (2.3)	1729 (27.0)
First hematocrit count, %			
≤35	694 (15.0)	367 (20.7)	1061 (16.5)
>35 and ≤40	1164 (25.1)	664 (37.5)	1828 (28.5)
>40 and ≤45	851 (18.3)	537 (30.4)	1388 (21.7)
>45	240 (5.2)	161 (9.1)	401 (6.3)
No labs/no record	1690 (36.4)	41 (2.3)	1731 (27.0)
First hemoglobin count, g/dL			
≤7	22 (0.5)	7 (0.4)	29 (0.5)
>7 and ≤10.4	341 (7.3)	172 (9.7)	513 (8.0)
>10.4 and ≤17.1	2538 (54.7)	1514 (85.5)	4052 (63.2)
>17.1	18 (0.4)	19 (1.1)	37 (0.6)
No labs/no record	1720 (37.1)	58 (3.3)	1778 (27.7)

*CT*, computed tomography; *ED*, emergency department.

**Table 2:** Characteristics of emergency physicians who saw sampled patient visits.

Characteristic (n [%])	n=43
Physician gender	
Male	23 (53.5)
Female	20 (46.5)
Years since completing medical school	
≤5 yrs	8 (18.6)
>5 and ≤10	13 (30.2)
>10 and ≤21	16 (37.2)
>21 and ≤35	6 (14.0)
Fellowship?	
No fellowship	33 (76.7)
Completed a fellowship	10 (23.3)
Annual visit volume for NTAP	
<95	9 (20.9)
95–124	11 (25.6)
125–204	11 (25.6)
≥205	12 (27.9)
% of CT among annual visits for each physician (n [%])	
≤ 10%	2 (4.7)
>10% and ≤20%	2 (4.7)
>20% and ≤25%	12 (27.9)
>25% and ≤30%	14 (32.5)
>30% and ≤30%	5 (11.6)
>35% and ≤40%	6 (13.9)
> 40 %	2 (4.7)

*NTAP*, non-traumatic abdominal pain; *CT*, computed tomography.

**Table 3:** Patient-visit characteristics and computed tomography (CT) ordering odds ratios (ORs).

	Univariate model		Multivariate model	
		
Characteristic	Unadjusted OR	P value	Adjusted OR	P value
Gender				
Male	Reference		Reference	
Female	0.83 (0.72, 0.96)	0.01	0.99 (0.85, 1.16)	0.95
Age				
18–22 yrs	0.33 (0.26, 0.43)	< 0.001	0.64 (0.52, 0.80)	< 0.001
23–30 yrs	0.51 (0.42, 0.61)	< 0.001	0.74 (0.60, 0.91)	0.005
31–43 yrs	0.73 (0.64, 0.82)	< 0.001	0.84 (0.73, 0.97)	0.02
44–63 yrs	Reference		Reference	
64–74 yrs	1.58 (1.29, 1.93)	< 0.001	1.39 (1.12, 1.73)	0.003
≥ 75 yrs	1.49 (1.23, 1.79)	< 0.001	1.35 (1.10, 1.66)	0.004
Arrival mode				
Walk-in	Reference		Reference	
Ambulance	1.11 (0.95, 1.30)	0.17	0.75 (0.65, 0.87)	<0.001
Indeterminate	0.41 (0.10, 1.68)	0.22	0.33 (0.07, 1.56)	0.16
Acuity (Emergency Severity Index)				
Most/more severe	6.75 (5.05, 9.04)	< 0.001	0.87 (0.59, 1.29)	0.48
Severe	5.40 (3.99, 7.31)	< 0.001	1.15 (0.81, 1.63)	0.45
Least/less severe	Reference		Reference	
No record	5.83 (3.15, 10.76)	< 0.001	1.500 (0.68, 3.31)	0.32
Arrival time				
Monday–Friday daytime	Reference		Reference	
Monday–Friday evening	0.98 (0.79, 1.22)	0.86	1.08 (0.89, 1.33)	0.43
Monday–Friday nighttime	1.03 (0.80, 1.33)	0.82	1.01 (0.80, 1.28)	0.95
Saturday–Sunday daytime	0.95 (0.79, 1.16)	0.63	1.00 (0.84, 1.19)	0.96
Saturday–Sunday evening	0.99 (0.79, 1.26)	0.97	1.14 (0.92, 1.40)	0.24
Saturday–Sunday nighttime	0.88 (0.71, 1.09)	0.25	0.83 (0.65, 1.06)	0.13
Disposition of patient visit				
Discharge	0.34 (0.28, 0.42)	< 0.001	1.21 (0.65, 2.23)	0.55
Admit	Reference		Reference	
Observation	1.07 (0.77, 1.48)	0.69	1.25 (0.91, 1.73)	0.17
Against medical advice/absent without leave/left without being seen	0.09 (0.04, 0.20)	< 0.001	0.48 (0.16, 1.51)	0.21
Indeterminate	0.49 (0.18, 1.36)	0.17	0.67 (0.24, 1.87)	0.44
Admission team				
Non-surgical team	Reference		Reference	
Surgical team	1.96 (1.55, 2.49)	< 0.001	1.84 (1.43, 2.37)	< 0.001
Not admitted	0.40 (0.33, 0.49)	< 0.001	0.67 (0.35, 1.29)	0.23
Advanced triage physician?				
No/indeterminate advanced triage	Reference		Reference	
Advanced triage	1.26 (1.09, 1.46)	0.002	0.97 (0.82, 1.15)	0.74
Prior CT abdomen/pelvis				
No	Reference		Reference	
Yes	0.63 (0.44, 0.91)	0.01	0.44 (0.30, 0.65)	< 0.001
ED arrivals in previous 4 hours				
≤42	1.10 (0.90, 1.36)	0.35	1.09 (0.83, 1.42)	0.54
>42 and ≤62	0.98 (0.80, 1.19)	0.83	0.97 (0.77, 1.24)	0.83
>62 and ≤79	Reference		Reference	
>79	0.95 (0.80, 1.14)	0.59	0.82 (0.68, 0.99)	0.04
Ultrasound abdomen/pelvis evaluation				
No	Reference		Reference	
Yes	0.93 (0.77, 1.13)	0.46	0.71 (0.58, 0.87)	< 0.001
First white blood cell count, K/mm3				
≤3.9	Reference		Reference	
>3.9 and ≤12.5	1.09 (0.82, 1.44)	0.56	1.06 (0.80, 1.42)	0.67
>12.5 and ≤15.5	2.51 (1.76, 3.58)	< 0.001	2.33 (1.61, 3.38)	< 0.001
>15.5	2.67 (1.96, 3.65)	< 0.001	2.24 (1.66, 3.03)	< 0.001
No labs/no record	0.05 (0.03, 0.08)	< 0.001	0.03 (0.002, 0.71)	0.03
First hematocrit count, %				
≤35	Reference		Reference	
<35 and ≤40	1.08 (0.91, 1.28)	0.40	1.00 (0.79, 1.25)	0.97
<40 and ≤45	1.19 (0.99, 1.44)	0.07	1.06 (0.87, 1.29)	0.55
>45	1.27 (1.03, 1.56)	0.02	1.07 (0.86, 1.33)	0.54
No labs/no record	0.05 (0.03, 0.07)	< 0.001	2.03 (0.12, 35.08)	0.63
First hemoglobin count, g/dL				
≤7	Reference		Reference	
>7 and ≤10.4	1.59 (0.67, 3.76)	0.30	1.75 (0.63, 4.88)	0.28
>10.4 and ≤17.1	1.87 (0.77, 4.57)	0.17	2.17 (0.78, 6.08)	0.14
>17.1	3.32 (1.08, 10.16)	0.04	3.26 (1.09, 9.74)	0.03
No labs/no record	0.11 (0.05, 0.24)	< 0.001	1.89 (0.52, 6.89)	0.34

*ED*, emergency department; *OR*, odds ratio.

**Table 4:** Physician characteristics and computed tomography (CT) ordering odds ratios (ORs).

	Univariate model		Multivariate model	
		
Characteristic	Unadjusted OR	P value	Adjusted OR	P value
Physician gender				
Male	Reference		Reference	
Female	1.23 (0.93, 1.63)	0.14	1.23 (0.93, 1.64)	0.15
Years since completing medical school				
≤5 yrs	0.89 (0.68, 1.17)	0.41	0.82 (0.63, 1.08)	0.16
>5 and ≤10	1.01 (0.73, 1.40)	0.93	0.99 (0.74, 1.32)	0.95
>10 and ≤21	Reference		Reference	
>21 and ≤35	0.55 (0.31, 0.96)	0.04	0.60 (0.39, 0.93)	0.02
Fellowship?				
No fellowship	Reference		Reference	
Completed a fellowship	0.62 (0.45, 0.85)	0.003	0.70 (0.53, 0.92)	0.01
Annual visit volume for NTAP				
<95	1.02 (0.75, 1.38)	0.91	1.19 (0.86, 1.65)	0.30
95–124	Reference		Reference	
125–204	0.93 (0.72, 1.19)	0.54	1.04 (0.76, 1.43)	0.82
≥205	1.11 (0.80, 1.54)	0.53	1.18 (0.87, 1.61)	0.28

*NTAP*, non-traumatic abdominal pain.

**Table 5:** Results of fixed effects from the multilevel model.

Variable type	Variable name	F value	Adjusted OR	P value
Patient-visit	Patient’s gender	0.03		
	Male		Reference	
	Female		0.99 (0.86, 1.14)	0.87
	Age	9.64		
	18–22 yrs		0.65 (0.50, 0.86)	0.003
	23–30 yrs		0.73 (0.60, 0.89)	0.002
	31–43 yrs		0.84 (0.71, 0.99)	0.04
	44–63 yrs		Reference	
	64–74 yrs		1.42 (1.15, 1.76)	0.001
	≥ 75 yrs		1.37 (1.10, 1.71)	0.006
	Arrival mode	7.90		
	Walk-in		Reference	
	Ambulance		0.75 (0.65, 0.88)	<0.001
	Indeterminate		0.31 (0.06, 1.59)	0.16
	Acuity (Emergency Severity Index)	3.89		
	Most/more severe		0.82 (0.57, 1.18)	0.29
	Severe		1.08 (0.78, 1.51)	0.63
	Least/less severe		Reference	
	No record		1.48 (0.78, 2.81)	0.23
	Arrival time	0.90		
	Monday–Friday daytime		Reference	
	Monday–Friday evening		1.04 (0.88, 1.24)	0.64
	Monday–Friday nighttime		0.88 (0.70, 1.12)	0.30
	Saturday–Sunday daytime		0.99 (0.78, 1.25)	0.91
	Saturday–Sunday evening		1.12 (0.88, 1.42)	0.37
	Saturday–Sunday nighttime		0.79 (0.58, 1.09)	0.15
	Disposition of patient visit	2.30		
	Discharge		1.17 (0.65, 2.09)	0.60
	Admit		Reference	
	Observation		1.21 (0.89, 1.64)	0.23
	Against medical advice/absent without leave/left without being seen		0.41 (0.15, 1.08)	0.07
	Indeterminate		0.68 (0.19, 2.39)	0.54
	Admission team	16.76		
	Non-surgical team		Reference	
	Surgical Team		1.88 (1.49, 2.38)	<0.001
	Not admitted		0.71 (0.39, 1.27)	0.24
	Advanced triage physician?	0		
	No/indeterminate advanced triage		Reference	
	Advanced triage		0.99 (0.82, 1.21)	0.95
	Prior CT abdomen/pelvis	12..34		
	No		Reference	
	Yes		0.43 (0.27, 0.70)	0.001
	ED arrivals in previous 4 hours	2.73		
	≤42		1.10 (0.88, 1.37)	0.39
	>42 and ≤62		0.98 (0.81, 1.18)	0.82
	>62 and ≤79		Reference	
	>79		0.80 (0.66, 0.97)	0.02
	Ultrasound abdomen/pelvis evaluation	12.59		
	No		Reference	
	Yes		0.70 (0.58, 0.86)	0.001
	First white blood cell count, K/mm3	19.04		
	≤3.9		Reference	
	>3.9 and ≤12.5		1.05 (0.77, 1.43)	0.77
	>12.5 and ≤15.5		2.28 (1.57, 3.32)	<0.001
	>15.5		2.25 (1.52, 3.33)	<0.001
	No labs/no record		0.03 (0.002, 0.62)	0.02
	First hematocrit count, %	0.33		
	≤35		Reference	
	>35 and ≤40		0.98 (0.79, 1.21)	0.86
	>40 and ≤45		1.06 (0.84, 1.32)	0.62
	>45		1.06 (0.78, 1.44)	0.69
	No labs/no record		2.11 (0.13, 35.44)	0.60
	First hemoglobin count, g/dL	1.57		
	≤7		Reference	
	>7 and ≤10.4		1.75 (0.69, 4.44)	0.24
	>10.4 and ≤17.1		2.19 (0.86, 5.57)	0.10
	>17.1		3.33 (1.01, 10.9)	0.047
	No labs/no record		1.87 (0.62, 5.68)	0.27
Physician	Physician gender	0.46		
	Male		Reference	
	Female		1.08 (0.86, 1.36)	0.50
	Years since completing medical school	2.22		
	≤5 yrs		0.89 (0.65, 1.20)	0.43
	>5 and ≤10		1.03 (0.82, 1.30)	0.79
	>10 and ≤21		Reference	
	>21 and ≤35		0.68 (0.48, 0.96)	0.03
	Fellowship?	1.39		
	No fellowship		Reference	
	Completed a fellowship		0.85 (0.65, 1.12)	0.25
	Annual visit volume for NTAP	1.86		
	<95		1.18 (0.85, 1.65)	0.31
	95–124		Reference	
	125–204		0.97 (0.71, 1.34)	0.87
	≥205		1.28 (0.97, 1.69)	0.08

*OR*, odds ratio; *CT*, computed tomography.

*OR*, odds ratio; *ED*, emergency department; *NTAP*, non-traumatic abdominal pain.

**Table 6:** Statistically significant results of fixed effects from the multilevel model.

Variable type	Variable name	F value	Adjusted OR	P value
Patient-visit	Age	9.64		
	18–22 yrs		0.65 (0.50, 0.86)	0.003
	23–30 yrs		0.73 (0.60, 0.89)	0.002
	31–43 yrs		0.84 (0.71, 0.99)	0.04
	44–63 yrs		Reference	
	64–74 yrs		1.42 (1.15, 1.76)	0.001
	≥ 75 yrs		1.37 (1.10, 1.71)	0.006
	Arrival mode	7.90		
	Walk-in		Reference	
	Ambulance		0.75 (0.65, 0.88)	<0.001
	Indeterminate		0.31 (0.06, 1.59)	0.16
	Admission team	16.76		
	Non-surgical team		Reference	
	Surgical team		1.88 (1.49, 2.38)	<0.001
	Not admitted		0.71 (0.39, 1.27)	0.24
	Prior CT abdomen/pelvis	12..34		
	No		Reference	
	Yes		0.43 (0.27, 0.70)	0.001
	ED arrivals in previous 4 hours	2.73		
	≤42		1.10 (0.88, 1.37)	0.39
	>42 and ≤62		0.98 (0.81, 1.18)	0.82
	>62 and ≤79		Reference	
	>79		0.80 (0.66, 0.97)	0.02
	Ultrasound abdomen/pelvis evaluation	12.59		
	No		Reference	
	Yes		0.70 (0.58, 0.86)	0.001
	First white blood cell count, K/mm3	19.04		
	≤3.9		Reference	
	>3.9 and ≤12.5		1.05 (0.77, 1.43)	0.77
	>12.5 and ≤15.5		2.28 (1.57, 3.32)	<0.001
	>15.5		2.25 (1.52, 3.33)	<0.001
	No labs/no record		0.03 (0.002, 0.62)	0.02
Physician	Years since completing medical school	2.22		
	≤5 yrs		0.89 (0.65, 1.20)	0.43
	>5 and ≤10		1.03 (0.82, 1.30)	0.79
	>10 and ≤21		Reference	
	>21 and ≤35		0.68 (0.48, 0.96)	0.03

*OR*, odds ratio; *CT*, computed tomography; *ED*, emergency department.
